# Quantification of metal‐induced susceptibility artifacts associated with ultrahigh‐field magnetic resonance imaging of spinal implants

**DOI:** 10.1002/jsp2.1064

**Published:** 2019-08-16

**Authors:** Yusuke Chiba, Hideki Murakami, Makoto Sasaki, Hirooki Endo, Daisuke Yamabe, Daichi Kinno, Minoru Doita

**Affiliations:** ^1^ Department of Orthopedics, School of Medicine Iwate Medical University Morioka Japan; ^2^ Division of Ultrahigh Field MRI, Institute of Biomedical Sciences Iwate Medical University Morioka Japan

**Keywords:** 7 T, artifact, metallic spinal implants, zero echo time

## Abstract

Reports on spinal‐implant metallic artifacts in 7‐T magnetic resonance imaging (MRI) are lacking. Thus, we investigated the magnitude of metal artifacts derived from spinal implants in 7‐T MRI and analyzed the differences obtained with spinal rods manufactured from pure titanium, titanium alloy, and cobalt‐chrome (5.5‐mm and 6.0‐mm diameters and 50‐mm length). Following the American Society for Testing and Materials guidelines, we measured the artifact size and artifact volume ratio of each rod during image acquisition using 7‐T MRI scanners with three‐dimensional (3D) T1‐weighted and 3D T2* spoiled gradient echo (GRE), 3D T2‐weighted fast spin echo, zero echo time (ZTE), and diffusion‐weighted imaging sequences. Pure titanium and titanium alloy rods yielded significantly smaller artifacts than did cobalt‐chrome rods, with no significant difference between pure titanium and titanium alloy rods. The artifact sizes of the 5.5‐mm and 6.0‐mm diameter rods were similar. The artifact magnitude increased in the following sequence order: ZTE, 3D T2 fast spin echo, 3D T1 spoiled GRE, 3D T2* spoiled GRE, and diffusion‐weighted imaging. Artifacts obtained using the spin echo method were smaller than those obtained with the GRE method. Because the echo time in ZTE is extremely short, the occurrence of artifacts because of image distortion and signal loss caused by differences in magnetic susceptibility is minimal, resulting in the smallest artifacts. ZTE can be a clinically useful method for the postoperative evaluation of patients after instrumentation surgery, even with 7‐T MRI.

## 1 INTRODUCTION

The role of spinal instrumentation surgery has increased in recent years and has greatly contributed to the improvement of surgical results. However, in terms of postoperative magnetic resonance imaging (MRI) examination of patients who have undergone instrumentation surgery, there is a concern about safety because of the presence of metal implants and the occurrence of artifacts. Although 0.5‐T to 3‐T MRI is clinically used in many cases and 7‐T MRI is currently limited to basic research, 7‐T MRI can provide high resolution images, as the signal‐noise intensity ratio also increases.^1^ Kraff produced a prototype 7‐T MRI and demonstrated in vivo images of the spine region of volunteers.^2^ He reported that the in vivo images demonstrated very fine anatomic features such as the longitudinal ligaments or the venous drainage through the vertebral bodies. Thus, clinical use of 7‐T MRI is expected to become more widespread in the future.

In our previous study, we investigated whether the displacement forces caused by a static magnetic field and the heating induced by radiofrequency (RF) radiation are substantial for spinal implants in a 7‐T field and reported on its safety^3^ in accordance with the American Society for Testing and Materials (ASTM), which sets MRI compatibility standards.^4^

Notably, however, higher magnetic fields tend to increase artifacts caused by metallic implants,^5^ and this phenomenon can compromise diagnostic accuracy. Some 3‐T MRI studies have suggested that the largest artifacts pertaining to spinal implants are associated with cobalt‐chrome, followed by titanium alloy and pure titanium. Artifacts associated with the gradient echo (GRE) method are reportedly larger than those associated with the spin echo (SE) method. Accordingly, similar results are expected for 7‐T MRI, but experimental evidence is lacking. Therefore, it is necessary to closely investigate the magnitude of artifacts in assessments that consider different metal compositions and imaging conditions.

We here quantitatively evaluated the magnitude of metal artifacts derived from spinal implants on 7‐T MRI and analyzed the differences between these artifacts based on the ASTM evaluation method.^6^ In addition, the zero echo time (ZTE) technique,^7^, ^8^, ^9^, ^10^ which is based on the three‐dimensional (3D) radial technique using dedicated software and RF coil technology equipped with ultrahigh‐speed RF switching, has gained attention in recent years. It has been suggested that this method can reduce acoustic noise and motion artifacts. To date, there has been no report on the reduction of metal artifacts, as far as we are aware, but generally, metal artifacts become smaller as the echo time (TE) shortens. Because TE is close to 0 in ZTE, the artifact is also expected to be very small; an artifact attenuation effect could be helpful in 7 T, where the artifact is relatively large originally. Thus, we expected that ZTE should reduce metal artifacts especially in 7‐T MRI. Therefore, we investigated ZTE in addition to the conventional sequences.

## 2 METHODS

### 2.1 Metal spinal implants

Six different metal rods that are frequently used in spinal surgery in clinical practice were examined. The rods were manufactured from either of three types of metals: pure titanium, a titanium alloy, or cobalt‐chrome (CD HORIZON SOLERA Spinal System, Medtronic, Minneapolis, Minnesota), with diameters of 5.5 or 6.0 mm; the length of all rods was 50 mm.

### 2.2 Evaluation of MR image artifacts

**Figure 1:**
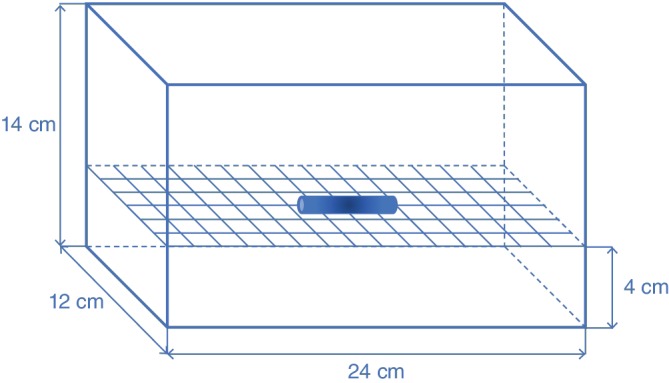
Schematic illustration of the phantom. The container of the phantom is composed of acrylic and is filled with vegetable oil. The container size is 24 × 14 × 12 cm. Each metal implant was placed at the center of a nylon net within the phantom

In accordance with the standard testing method for the evaluation of MRI artifacts from passive implants provided by the ASTM,^5^ we measured artifact size and the volume ratio of each metal spinal implant during image acquisition using a 7‐T MRI scanner (Discovery MR950, GE Healthcare, Milwaukee, Wisconsin) with quadrature transmission and a 32‐receiver head coil (NM008‐32‐7 GE‐MR950; Nova Medical, Wilmington, Massachusetts). Each metal implant was placed on a nylon net in an acrylic container (Figure 1) filled with vegetable oil to produce a uniform signal and intensity of the phantom at 7 T.^11^ Nylon is very insensitive to magnetic forces, and therefore, causes less artifacts. ASTM also recommends the use of nylon because it does not cause distortion.^6^ For the same reason, acrylic is often used in many similar studies.^12^, ^13^

The following imaging sequence was used: 3D T1‐weighted spoiled GRE (3D T1 SPGR), 3D T2*‐weighted SPGR (3D T2* SPGR), 3D T2‐weighted fast SE (3D T2 FSE), ZTE, and diffusion‐weighted SE‐planar imaging (DWI). The scanning parameters are shown in Table 1.

**Table 1:** The scanning parameters of each sequences

	3D T1 SPGR	3D T2* SPGR	3D T2 FSE	ZTE	DWI
TR (ms)	6.3	30	3000	50	10 000
TE (ms)	1.8	15	73	0.016	76.9
FA (degree)	15	20	90 / 180	4	90 / 180
Band width (kHz)	41.7	31.3	83.3	32	256
Matrix size (frequency/phase)	256 × 256	256 × 256	256 × 256	128 × 128	64 × 128
Slice thickness (mm)	1	1	1	2	2
Number of slices	132	132	132	128	64
Acquisition time (s)	104	407	355	293	20

*Note*: Field of view, 25.6 cm; reconstruction image matrix, 512 × 512. Sagittal to static field direction image were acquired in all case.

Abbreviations: 3D T1 SPGR, 3D T1 weighted spoiled gradient echo; 3D T2 FSE, 3D T2 weighted fast spin echo; 3D T2* SPGR, 3D T2* weighted SPGR; DWI, Diffusion‐weighted spin echo echo‐planar imaging; ZTE, zero echo time.

The rods were placed in the phantom in directions parallel and perpendicular to the static field (B0) of the magnet center. Sagittal MRI in the B0 direction with swapping phase and frequency encoding were obtained with and without the rod. The artifact areas were defined as areas showing a signal intensity that differed by more than 30% between the conditions with and without the rod, according to the ASTM guidelines. The artifact image was generated using the subtraction method via paired images, with and without the rod, using the same direction and frequency encoding in each sequence.

To determine the artifact size, a line region was defined across the center of the artifact area in each artifact image, and a signal‐intensity profile was generated via ImageJ (National Institutes of Health, Bethesda, Maryland). The number of pixels counted in the line region was converted to a measurement of distance (mm). Artifact distances of the rod were calculated using the following equations: (Artifact distance in long axis direction − long diameter of the rod)/2 or (Artifact distance in short axis direction − short diameter of the rod)/2. To determine the artifact volume, the total number of artifact pixels in all regions was determined from the artifact images. The number of artifact pixels counted was converted into total artifact volume (mm^3^). Thereafter, the artifact volume ratio (total artifact volumes/rod volume) was calculated for each imaging sequence.

### 2.3 Statistical analyses

The Wilcoxon signed‐rank test, with and without Bonferroni correction, was used to analyze differences between rod materials, rod diameter, rod installation direction, and frequency direction. The Steel‐Dwass test was used to analyze imaging sequences. The alpha level used was 0.05. We used SPSS 24 for Mac (SPSS Inc., Chicago, Illinois) and R 3.5.1 (R Foundation for Statistical Computing, Vienna, Austria) for statistical analysis.

## 3 RESULTS

**Figure 2:**
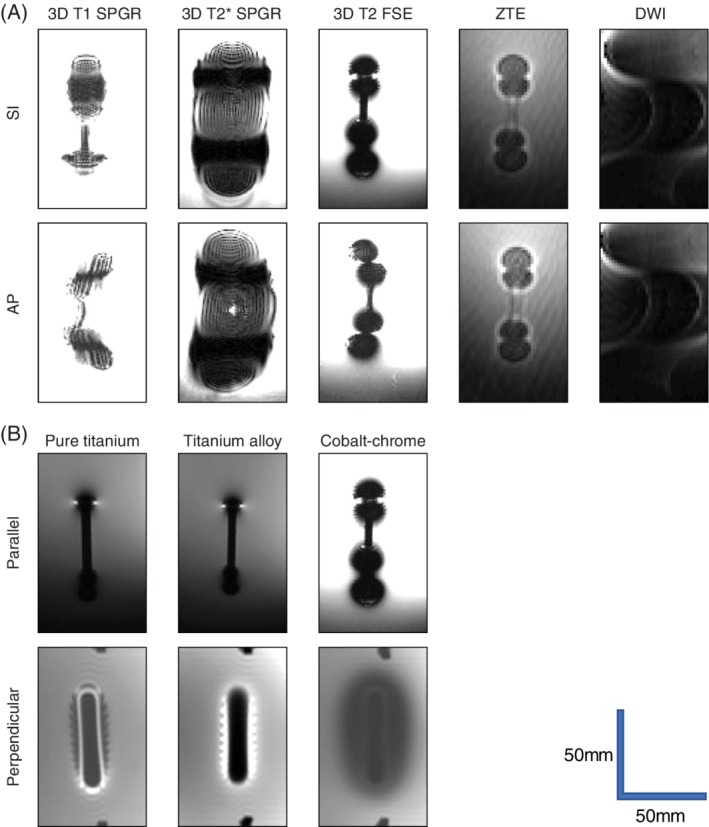
Typical magnetic resonance images of the rods. A, A cobalt–chrome rod of 6 mm in diameter and 50 mm in length was placed parallel to the static magnetic field. The appearance of artifacts differed for each sequence and frequency encoding direction. B, Images of a rod with a diameter of 6 mm obtained with 3D T2‐weighted fast spin echo and a frequency encoding direction of superior–inferior. The appearance of artifacts differed for each material and for each rod installation direction (with respect to the static magnetic field). When the rod is located parallel to the static magnetic field, the image is shown as sagittal, and when the rod is located perpendicular to the static magnetic field, the image is shown as coronal

DWI did not yield accurate measurements of the artifact area because the artifact area exceeded the measurable range in all rods. Then, we compared and examined the artifacts obtained with the following four sequences; 3D T1 SPGR, 3D T2* SPGR, 3D T2 FSE, and ZTE. Figure 2 shows typical MRI of the rods. The median and quartile range of the measured data are shown in Table 2.

**Table 2:** Artifact data performed in 7‐T Field

Material	Length of artifact (mm)		Volume ratio	
Pure titanium	Median	IQR	Median	IQR
5.5 mm	12.2	9.9	14.0	39.4
6.0 mm	13.0	9.5	13.8	39.2
Titanium alloy				
5.5 mm	13.2	12.9	14.9	42.1
6.0 mm	12.8	13.1	14.1	40.9
Cobalt‐chrome				
5.5 mm	20.2	13.7	45.1	66.7
6.0 mm	20.4	10.4	44.0	55.3
Pure titanium				
SI	13.0	10.9	14.6	39.4
AP	12.7	10.1	13.8	42.5
Titanium alloy				
SI	12.6	11.8	15.5	42.0
AP	13.7	12.6	14.1	44.6
Cobalt‐chrome				
SI	23.4	18.4	47.3	65.9
AP	19.5	13.0	51.5	66.6
Pure titanium				
Parallel	11.4	10.4	5.6	38.2
Perpendicular	13.4	10.5	23.0	72.4
Titanium alloy				
Parallel	11.7	10.9	5.8	39.3
Perpendicular	13.7	12.8	22.7	75.9
Cobalt‐chrome				
Parallel	18.1	9.5	38.1	39.1
Perpendicular	20.9	15.2	86.6	67.8
Pure titanium				
3D T2 FSE	9.1	6.3	11.1	16.9
3D T1 SPGR	13.4	4.0	17.1	17.4
3D T2* SPGR	21.8	4.9	79.3	55.8
ZTE	7.9	4.9	4.5	7.9
Titanium alloy				
3D T2 FSE	8.4	6.5	11.4	16.5
3D T1 SPGR	15.6	7.1	17.8	20.4
3D T2* SPGR	21.1	4.9	82.0	51.9
ZTE	7.5	4.8	4.2	7.6
Cobalt‐chrome				
3D T2 FSE	21.0	13.0	71.7	45.4
3D T1 SPGR	22.5	8.0	60.7	52.0
3D T2* SPGR	NM		NM	
ZTE	14.4	7.2	16.7	16.9

Abbreviations: AP, anterior–posterior; IQR, interquatile range; NM, not measurable; SI, superior–inferior.

### 3.1 Length of the artifacts

With respect to rod materials, the pure titanium and titanium alloy rods yielded significantly lower artifact length than did the cobalt‐chrome rods (*P* < 0.01, Wilcoxon signed‐rank test, with Bonferroni correction); however, there was no significant difference between the pure titanium and titanium alloy rods.

In terms of diameters, rods with a diameter of 6.0 mm showed significantly larger artifacts than those with a diameter of 5.5 mm for pure titanium rods, but there was no difference between the different diameters for the titanium alloy and cobalt‐chrome rods (*P* = 0.067, 0.103, respectively; Wilcoxon signed‐rank test).

By installation direction, the artifact size was larger when the titanium alloy rod was installed perpendicular rather than parallel to the static magnetic field direction (*P* < 0.05, Wilcoxon signed‐rank test). On the other hand, there was no difference according to installation direction for the pure titanium and cobalt‐chrome rods(*P* = 0.112, 0.086 Wilcoxon signed rank test). There was no significant difference in the frequency direction for all materials (*P* = 0.071‐0.936, Wilcoxon signed‐rank test).

**Figure 3:**
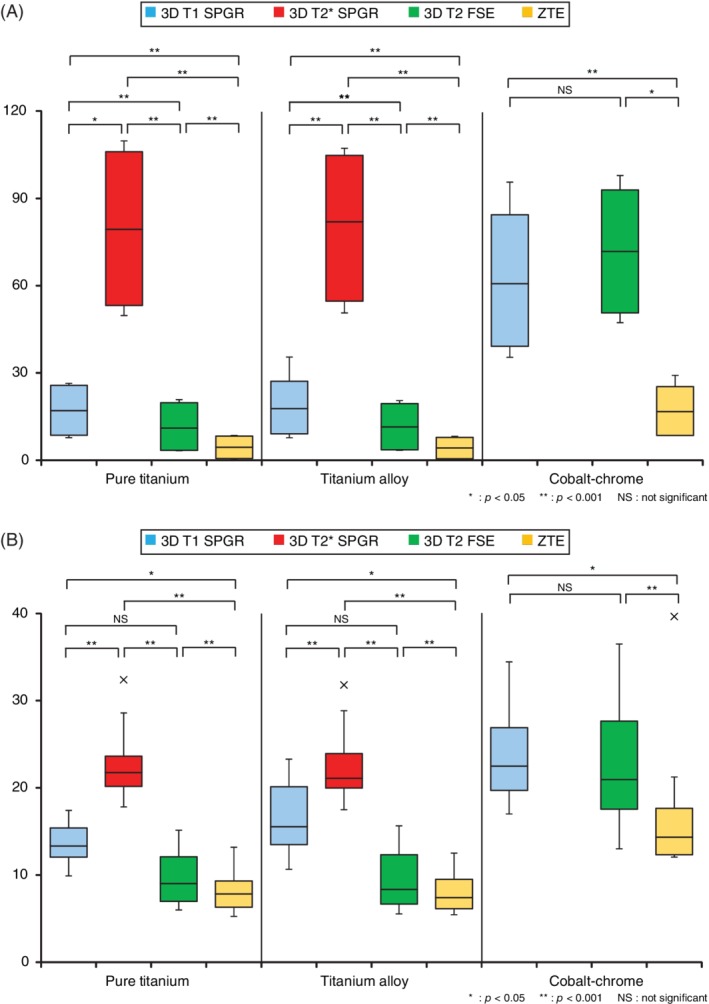
The magnitude of the artifact for each imaging sequence. A, Artifact distance of each sequence. Artifacts tended to be smaller in the order of zero echo time (ZTE) > T2 > T1 > T2*. B, Artifact volume ratio for each sequence. Artifacts tended to be smaller in the order of ZTE > T2 > T1 > T2*

Comparisons of the length of the artifacts according to the sequence used yielded the following hierarchy: ZTE = 3D T2 FSE < 3D T1 SPGR <3D T2* SPGR for the titanium alloy and pure titanium rods (Steel‐Dwass test) (Figure 3A). For the cobalt‐chrome rods, ZTE yielded significantly smaller artifacts than did 3D T1 SPGR and 3D T2 FSE; there was no significant difference between 3D T1 SPGR and 3D T2 FSE. 3D T2* SPGR yielded artifacts beyond the imaging range, making measurement difficult for cobalt‐chrome rods (Steel‐Dwass test) (Figure 3A).

### 3.2 Artifact volume ratio

There were no significant differences associated with rod diameter (*P* = 0.182‐0.836, Wilcoxon signed‐rank test) or frequency direction (*P* = 0.326–0.717, Wilcoxon signed‐rank test). Artifact volume ratios were smaller when the direction of rod installation was parallel rather than perpendicular to the static magnetic field direction (*P* < 0.01, Wilcoxon signed‐rank test). With respect to sequence, for pure titanium and titanium alloy rods, there was no significant difference between the ZTE and 3D T2 FSE, 3D T2 FSE, and 3D T1 SPGR sequences, but ZTE yielded significantly smaller artifacts than did 3D T1 SPGR. Additionally, 3D T2* SPGR yielded significantly larger artifacts than did the other sequences. On the other hand, for cobalt‐chrome rods, ZTE yielded significantly smaller artifacts than did 3D T1 SPGR and 3D T2 FSE, and there was no significant difference between the 3D T1 SPGR and 3D T2 FSE sequences. As with the artifact distance results, 3D T2* SPGR yielded artifacts beyond the imaging range, making measurement difficult for cobalt‐chrome rods (Steel‐Dwass test) (Table 2, Figure 3B).

## 4 DISCUSSION

MRI produces images by emphasizing the density and physical properties (T1, T2 value, etc.) of hydrogen atoms (protons) by implementing the nuclear magnetic resonance phenomenon. If magnetic susceptibility varies greatly in the living body, the non‐uniformity of the static magnetic field becomes large and the frequency distribution shifts. As a result, intravoxel phase distortions occur before echoes are regenerated, causing image artifacts due to image distortion and signal loss. The most prominent artifacts occur when metal is present inside the body, such as after fixation using instrumentation in spinal surgery. As a measure of how much magnetization is present per unit in a static magnetic field, magnetic susceptibility is used, and the larger it is, the greater is the influence on the MR image. Thus, the influence of artifacts increases in a high magnetic field. In this basic study, we evaluated the metal artifacts of spinal implants in 7‐T MRI and investigated the possibility of image diagnosis after spinal fusion when 7‐T MRI is clinically introduced.

The ASTM has propounded a method for evaluating MR compatibility of medical equipment.^6^ They stipulated that the target object should be placed on a nylon net in an aqueous solution and should be evaluated using the maximum distance from the object to the boundary where the image signal intensity changes by 30%, and that both the GRE and SE methods should be used. We performed our evaluations according to these guidelines. Additionally, the ASTM recommends using an aqueous copper sulfate solution for the phantom solution when installing metal rods. However, in this study, we used an ultrahigh magnetic field of 7 T. When an aqueous solution of copper sulfate is used, an image with a uniform signal intensity over a wide range cannot be obtained at this field strength due to interference of the transmitted electromagnetic waves; thus, this solution is not suitable for measuring the artifact range. Therefore, we used vegetable oil as a solution due to its low dielectric constant property. As imaging sequences, we used both the GRE and SE methods and the clinically‐frequently used DWI and ZTE sequences, which have gained increasing interest in recent years.

We demonstrated that pure titanium and titanium alloy produced significantly smaller artifacts than did cobalt‐chrome. Titanium alloy and cobalt‐chrome are currently widely used in spinal fixation surgery. Although titanium alloy is inferior in strength, it is considered to be suitable for postoperative follow‐up using MRI because it produces smaller artifacts due to its lower magnetic susceptibility. As reported for 1.5‐T,^14^, ^15^ we found that titanium alloy was also advantageous for reducing artifacts, even at 7 T.

In terms of rod diameters, there was a statistically significant difference in the distance of the artifact between pure titanium rods with a diameter of 5.5 mm and of 6.0 mm, but there was no significant difference between titanium alloy and cobalt‐chrome. The artifact volume ratio according to rod diameter did not differ significantly for any material and was visually equal for all. Previous reports using 1.5‐T MRI have suggested that a difference in rod diameter from 4.75 to 5.5 mm does not differentially affect artifact production.^16^ Even at an ultra‐high magnetic field of 7 T, the influence of a 0.5‐mm difference in rod diameter, as used in this study, had a negligible effect on artifacts.

Regarding the magnitude of the artifacts according to rod installation direction, artifacts were significantly smaller when installed parallel rather than perpendicular to the static magnetic field direction. This is because artifacts differ in shape with respect to the static magnetic field direction, as previously reported for imaging of stents using 1.5‐T and 3‐T MRI^17^; we propose that the size of artifacts could be attenuated by placing them parallel with respect to the static magnetic field direction, even at 7 T. When 7‐T MRI is clinically implemented in the future, it is likely that pedicle screws and transverse connectors installed perpendicular to the trunk axis will produce more severe artifacts than rod‐shaped metal implants installed in parallel.

For each imaging sequence, the magnitude of the artifact decreased in the order of DWI > GRE method > SE method > ZTE. DWI is very susceptible to the influence of magnetic susceptibility,^18^ and artifacts exceeded the imaging range in this study. As previously reported, distortion is reduced when using an SE method because the frequency deviation is corrected with the 180° pulse for reconvergence. Therefore, as previously reported for 1.5‐T and 3‐T MRI,^19^ artifacts obtained using the SE method were smaller than those obtained using the GRE method for 7 T.

On the other hand, ZTE is an image generated from extremely short TE. By using short TE, the phase dispersion within the voxel is small and thus the magnetic susceptibility artifact is maintained. As a result, the artifact sizes in the ZTE image were smaller than those obtained with the SE methods.^20^ In particular, the volumes of artifacts in ZTE images of pure titanium and titanium alloy rods were smaller than those in images obtained with any rod under all other conditions. Holdsworth reports that ZTE T1‐weighted images have an equal or greater contrast compared with the conventional images.^21^ Alibek reports that ZTE T1‐weighted images achieved diagnostic image quality in brain MRI.^22^ With regard to the contrast of white matter, gray matter, and cerebrospinal fluid, an image quality equivalent to that obtained with the conventional method can be achieved. Therefore, it is considered that the image quality with the ZTE technique is better than that achieved with the conventional method around the spinal canal, and a reduction of artifacts is expected. Therefore, ZTE may be an effective method for evaluating the state of the spinal canal after spinal instrumentation surgery, even at 7 T.

This study had some limitations. First, since it was not an in vivo experiment, the artifacts in this study may be different from those obtained in the human body. 7‐T MRI in humans with metal in their bodies cannot be currently performed, and it will be necessary to reevaluate this after the safety of this technique has been established in the future. Second, because the 7‐T MRI scanner used in this study was a device dedicated to head imaging, there was no receiver coil for the trunk. Also, because ASTM guidelines require a distance of 4 cm from the edge of the container, we used a 50‐mm‐long rod, which is the longest size for use with the container in the head coil. In the future, the magnitude of artifacts obtained with long rods used in a wide range of fixation techniques will need to be confirmed when whole‐body imaging becomes possible with 7‐T MRI. In addition, it is time consuming to shoot all the sequences for all rods with different diameters and diverse material composition. Therefore, because of time constraints, we obtained images of each rod with each sequence only once, and the thus accuracy of measurements is a concern.

## 5 CONCLUSION

We measured and investigated spinal implant artifacts obtained when using various 7‐T MRI sequences, according to the ASTM guidelines. To the best of our knowledge, no published reports have so far described the evaluation of artifacts associated with spinal implants via 7‐T MRI. As this imaging modality is becoming more widely used, these findings will be of value. Additionally, we demonstrated that ZTE can be a clinically useful method for the postoperative evaluation of patients after instrumentation surgery, even using 7‐T MRI.

## CONFLICT OF INTEREST

All authors declare that there are no conflicts of interest pertaining to any company or entity related to this manuscript.

## AUTHOR CONTRIBUTIONS

Y.C. contributed to the research design and interpretation of the data. H.M. and M.S. drafted the manuscript. H.E. analyzed the data. D.Y., D.K., and M.D. provided advice and guidance for research. All authors have read and approved the final submitted manuscript.
